# A randomised controlled trial of patient led training in medical education: protocol

**DOI:** 10.1186/1472-6920-10-90

**Published:** 2010-12-03

**Authors:** Anna E Winterbottom, Vikram Jha, Colin Melville, Oliver Corrado, Jools Symons, David Torgerson, Ian Watt, John Wright

**Affiliations:** 1 Yorkshire Quality and Safety Research Group, Bradford Institute for Health Research, Bradford Royal Infirmary, Duckworth lane, Bradford, BD9 6RJ, UK; 2 Leeds Institute of Medical Education, Level 7, Worsley Building, University of Leeds, LS2 9JT, UK; 3 North Yorkshire and East Coast Foundation School, Yorkshire and the Humber Deanery East Locality, Building 1, Willerby Hill Business Park, Willerby, Hull, HU10 6FE, UK; 4 West Yorkshire Foundation School, Postgraduate Centre, Leeds General Infirmary, Great George Street, Leeds LS1 3EX, UK; 5 York Trials Unit, Department of Health Sciences, University of York, Heslington, York, YO10 5DD, UK; 6 Department of Health Sciences, Seebohm Rowntree Building, University of York, Heslington, York, UK. YO10 5DD, UK

## Abstract

**Background:**

Estimates suggest that approximately 1 in 10 patients admitted to hospital experience an adverse event resulting in harm. Methods to improve patient safety have concentrated on developing safer systems of care and promoting changes in professional behaviour. There is a growing international interest in the development of interventions that promote the role of patients preventing error, but limited evidence of effectiveness of such interventions. The present study aims to undertake a randomised controlled trial of patient-led teaching of junior doctors about patient safety.

**Methods/Design:**

A randomised cluster controlled trial will be conducted. The intervention will be incorporated into the mandatory training of junior doctors training programme on patient safety. The study will be conducted in the Yorkshire and Humber region in the North of England. Patients who have experienced a safety incident in the NHS will be recruited. Patients will be identified through National Patient Safety Champions and local Trust contacts. Patients will receive training and be supported to talk to small groups of trainees about their experiences. The primary aim of the patient-led teaching module is to increase the awareness of patient safety issues amongst doctors, allow reflection on their own attitudes towards safety and promote an optimal culture among the doctors to improve safety in practice. A mixture of qualitative and quantitative methods will be used to evaluate the impact of the intervention, using the Attitudes to Patient Safety Questionnaire (APSQ) as our primary quantitative outcome, as well as focus groups and semi-structured interviews.

**Discussion:**

The research team face a number of challenges in developing the intervention, including integrating a new method of teaching into an existing curriculum, facilitating effective patient involvement and identifying suitable outcome measures.

**Trial Registration:**

Current controlled Trials: ISRCTN94241579

## Background

Patient safety is an international health priority [1]. Estimates suggest that approximately 1 in 10 patients admitted to National Health Services hospitals in the UK is involved in an incident which results in harm to patients [2] with a subsequent cost to the NHS of over £2 billion [3]. Strategies to reduce patient safety incidents have shifted the focus from a person centred approach where errors are viewed as occurring as the result of the individual, to developing a systems approach whereby individual error is accepted and mechanisms are put in place to ensure that error is reduced in the environment in which it occurs [3,4].

There is an increasing international drive to involve patients in safety initiatives [5]. However recent reviews of the literature have highlighted: a lack of initiatives to promote patient and/or carer involvement in patient safety; major gaps in our knowledge about the nature and impact of patient involvement; little evidence of the feasibility or effectiveness of patient centred interventions and uncertainty over their acceptability amongst patients and health professionals [5,6]. One role for patients in improving patient safety is in teaching health professionals as part of educational interventions. Patient-led teaching for healthcare professionals has been reported to be effective in terms of learner satisfaction and improved performance in key areas such as communication skills [7]. Patient narratives involving patients sharing their stories with professionals are now widely employed as part of medical training and clinical skills acquisition [7,8]. Patient safety is a particularly appropriate topic for such teaching as patients can bring real lived experiences of error and harm to the classroom, describe the personal impact of such errors and facilitate discussion around the error. There is a paucity of research which examines the use of patient stories in a safety context; although preliminary research suggests that this is a feasible method for communicating concerns about patient safety to healthcare professionals [6]. The present study aims to develop an intervention based on patients as teachers in training junior doctors about patient safety and to evaluate the impact of this intervention on safety attitudes in a randomised controlled trial.

## Method

### Design

The study is a randomised controlled trial conducted in the North of England with a two step approach covering West and North/East Yorkshire.

### Setting

The study will be conducted in two postgraduate medical schools in the Yorkshire and Humber region, West Yorkshire (WY) and North East Yorkshire and North Lincolnshire (NEYNL). In West Yorkshire, teaching on patient safety takes place at two sites; in the trial one site will receive the intervention (Harrogate Hospital) and a second site will receive the control (Airedale Hospital). In NEYNL, teaching takes place at five sites (Scunthorpe, Scarborough, Hull, Grimsby and York Hospitals). There are ten teaching sessions in total with each of the hospitals being responsible for delivering some of the patient safety teaching. There is variation in the number of sessions provided at each site, e.g. Grimsby, n = 1; York, n = 3. Teaching at these sites commences in January 2011 for approximately 8 weeks. The first phase of the study in the West Yorkshire Foundation School will allow for the refinement of our methodology and serve as a trial for our outcome measures. In NEYNL Foundation School, the intervention and control arm will run in parallel sessions, trainees and participants will be randomly assigned to either group on the day of teaching.

### Context

The intervention will be incorporated into the mandatory training of the first year postgraduate training for patient safety, i.e. Foundation Year 1 (FY1) trainees.

### Sample

Two separate populations will be recruited a) patients and b) trainee doctors

#### a) Patients

Two types of patients will be recruited and will receive training to deliver the intervention. Firstly patient safety champions will be identified through a national network (National Patient Safety Agency (NPSA) and Action against Medical Accidents (AVMA)). This network of trained volunteers provides advocates of patient safety and a source of stories about the personal impact of error in diagnosis, treatment and care. Secondly, local patients will be identified from patient and public involvement contacts and existing patient safety research networks. There are 16 teaching sessions that require patient input. We therefore anticipate recruiting 4-5 local patient safety champions and 4-5 national patient champions to deliver this teaching in both regions. This will mean that each patient is not overburdened with the number of sessions they deliver or the time and travel required participating in the study.

#### b) Trainee doctors

**Figure 1:**
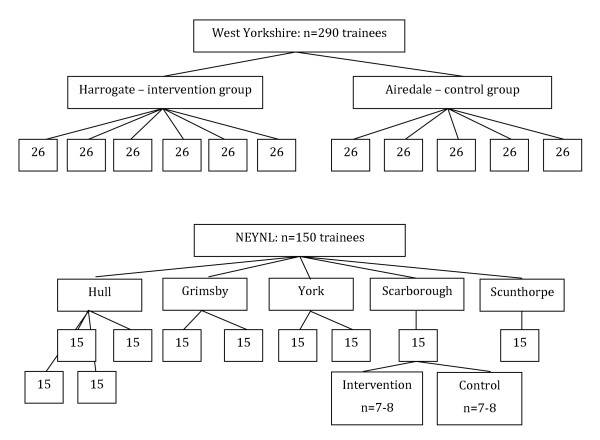
**Approximate number of participants assigned to each site**.

In West Yorkshire there are currently 290 FY1 trainees enrolled. Eleven teaching sessions are conducted with approximately 26 trainees attending each session (intervention or control). In NEYNL, approximately 150 trainees will be enrolled as FY1. Ten teaching sessions on patient safety are conducted; therefore approximately 15 trainees attend each session (Figure 1). Each session will be equally divided, with approximately 7-8 trainees receiving the control and 7-8 receiving the intervention, as illustrated with Scarborough in Figure 1.

### Sample Size calculation

In NEYNL, there are 150 trainees that will be allocated into 20 groups of 7-8 trainees. Assuming an intracluster correlation coefficient of 0.05 this gives a design effect of 1.3. The effective sample size, therefore, is 150/1.3, which is 115. This gives us approximately 80% power to demonstrate an effect size of 0.53.

### Ethical Approval

Ethical approval for the study was granted by the National Research Ethics Committee in February 2010.

### Procedure

Trainees in the control group will be informed that their course is being evaluated and they will be asked to complete questionnaires during their training. Trainees in the intervention group will receive the same information, and in addition, they will be informed that their teaching will consist of patients delivering stories about their experience of a safety incident. Using the patient experience is an established part of medical teaching and it is not anticipated that trainees will refuse to be taught in this manner. Those who do not wish to take part in either the control or intervention group will not be asked to complete questionnaires.

Participants in West Yorkshire will consent to the study at the beginning of the teaching session. At the intervention site (Harrogate) teaching will be delivered in the morning session (approximately three hours of teaching). Two patient champions will speak about their experiences which will be based around the three themes covered in the control group, namely, prescribing, teamwork and effective communication. The control group (Airedale) will receive three sessions also on these topics, communicated using the standard methods of teaching, which includes PowerPoint presentations and small group work. In the afternoon, at both sites, all trainees will receive training on: Personal organisation, Handover, Prioritisation and Transfusion. Questionnaires will be completed before the intervention and returned on the day of teaching; participants will also be required to complete the questionnaires at the end of the teaching session and one month later.

Trainees in NEYNL will be consented on the day of the study. Any trainees objecting to taking part in the intervention will be assigned to the control group, which will run in parallel to the intervention. The control will receive the standard teaching and the intervention will receive the intervention. These sessions will run simultaneously.

### Development of the intervention

All patient participants identified as potentially suitable for the study, will receive a letter of introduction, a study information sheet and provide written consent to participate in the research. Those who express an interest in the study will be invited to attend an open day where they can learn more about the research and decide whether or not they would like to take part. Preparatory patient learning journey (PLJ) workshops will be held for all patients participating in the training programme. These will be facilitated by members of the 'patient voice' group at the University of Leeds. These facilitators, who are patients and carers themselves, help to create a supportive learning environment where the patients feel comfortable about sharing their experiences and valued for their contribution. The approach focuses on three sessions:

a) The first session is on the 'patient journey' in which the patients are given the opportunity to think and talk about their own journey and share their experiences. This session particularly focuses on key contributory factors for safety such as hygiene, communication, medications and observations.

b) The second session allows patients to reflect on the content of the first session and discuss how things could have been done differently to improve their experiences with healthcare.

c) The third session builds on the first two sessions to allow patients to think about how their experiences and knowledge can help health professionals to learn and develop their own practice. In addition, it focuses on how these patients could be involved in teaching healthcare professionals. At the end of these sessions patients will have developed their experiences into a story of approximately 20 minutes in length that they will relay to trainees in the teaching session. The precise content of the talk is discussed within the patient voice group, but is largely decided upon by the patients themselves, e.g. the patients may choose to focus on one particular aspect of their experience, or supplement their talk with a PowerPoint presentation to clarify their message. To ensure patients are fully supported and feel confident they will be briefed before and after the teaching sessions. A third patient participant will also attend each teaching session to take observational notes and serve as a reserve, in case one of the patients is unable to attend.

### Intervention

The teaching session will use patient stories to focus both on the specific issues around the individual patient story as well as more generic issues around patient safety. Emphasis will be given to issues of analysis and causes of errors. The stories will include factual descriptions of what happened and reflections about their experience with medical error, what went wrong, why it took place, what the NHS response was, the impact of the error, the information they were given, what could be done better and why. The sessions will be interactive with questions and debate. Facilitation will be provided by a trained independent chairperson along with the patients. Participants will be asked to reflect on the patients' narrative, to identify key themes on patient safety emerging from the stories and to explore their own attitudes and beliefs towards patient safety. They will also be encouraged to share their own experiences of safety incidents, both as professionals and as patients or carers themselves.

### Measures

Measuring error is difficult [9]. It would be unrealistic to expect be able to detect or attribute changes in patient safety incident rates, for example through case note review, for junior doctor's practice as part of their multidisciplinary team. The primary aim of the patient-led teaching module is to facilitate awareness of patient safety issues, allow reflection on and facilitate changes to their attitudes to patient safety and above all, promote a safety culture among doctors at such an early stage of their training. It is hoped that this will translate into improved safety in practice.

Outcome measures for the study will combine quantitative and qualitative methods and include:

1. The Attitudes to Patient Safety Questionnaire (APSQ) [10], a reliable and validated 26-item questionnaire addressing patient safety attitudes. It was originally designed to target senior medical trainees which make it applicable to FY1 trainees at the start of their clinical practice.

2. Knowledge of patient safety will be measured using a reliable and validated 7 item measure specifically designed to target medical trainees [11]. Both measures will be administered to all trainees in both the intervention and control groups before and after the training session and 4-6 weeks later.

3. All trainees will be asked to complete a course evaluation at the end of the teaching session to assess overall effectiveness.

4. Twenty in depth interviews with a purposive sample of ten trainees from each group (control/intervention) will explore their views on the impact of medical error on patient outcome and the perceived usefulness and acceptability of the two training sessions. These will be conducted 4-6 weeks after the teaching session. Feedback from patients and experts suggests that patient stories provoke an emotional response in the learner. Trainees will be specifically asked about any psychological impact of the patient narratives on themselves with the intention of developing a measure to examine learning. Any differences in views on the perceived value and effectiveness of the two teaching sessions will be evaluated. The patients involved in the training will also be interviewed individually to explore their views on how effective the teaching programme was, how it had helped them understand the health system and reasons for medical errors. Transcripts will be content-analysed using thematic framework analysis. A constant comparative approach to the analysis of qualitative data will be used.

5. Observational notes will be taken by a third patient participant. The notes will focus on signs of audience engagement, levels of interest such as non verbal cues.

6. Trainees will be asked to identify up to 3 learning points that they identify at the end of the teaching session, that they intend to implement into practice. Trainees will be followed up at one month to see whether or not they have been successful in doing so.

### Analysis

We will undertake a regression based analysis adjusting for any clustering effects using the Huber-White approach as implemented in STATA. We will adjust for baseline test scores.

## Discussion

The strength of this research is our commitment to patient involvement. The patients in our study will not only deliver the intervention, but will be involved in defining the aims and objectives of the teaching sessions, provide feedback on documents, e.g. our interview guide for trainee doctors; and will take part in focus groups after the teaching sessions to refine the intervention. This will require considerable commitment from the patient and in recognition of their contribution they will be provided with financial reimbursement for their attendance on the teaching and training days, in addition to travel expenses.

This research also poses a number of challenges. Firstly, patient safety teaching is already established in both West Yorkshire and NEYNL. The research team must therefore negotiate the integration of the study into the existing teaching curriculum. This requires careful consideration of pre-existing learning outcomes to ensure that the teaching fulfils the Foundation School's obligation to provide comprehensive safety training. The research team must also work closely with Foundation Year 1 co-ordinators and facilitators to ensure successful implementation of the study. Secondly, there is little evidence of optimal objective outcome measures for educational interventions, particularly in the medium or long term. There is also a lack of consensus as to whether attitudes can be changed and whether these changes will necessarily translate into professional behaviour. To address this challenge we intend to use a variety of measures, i.e. questionnaires, interviews, focus groups and self reported learning to provide a richness of data.

## Competing interests

The authors declare that they have no competing interests.

## Authors' contributions

AW leads the research project and drafted the manuscript; VJ is Chief Investigator on the project, was co-applicant for grant funding, and contributed to design of the study and draft of manuscript; OC and CM are co-directors of Yorkshire Foundation schools and have provided the setting for the research and contributed to the design of the study; JS will prepare and support the patient participants to enable them to take part in the intervention. IW was co-applicant for grant funding, and contributed to design of the study; JW was lead applicant on for grant funding of the project and contributed to the design of the study. All authors are part of the core research team that meet regularly to discuss the design and implementation of the study; all members have read drafts and approved the final manuscript.

## Pre-publication history

The pre-publication history for this paper can be accessed here:

http://www.biomedcentral.com/1472-6920/10/90/prepub
